# Dislocation of a revision total knee arthroplasty: rare but serious complication

**DOI:** 10.11604/pamj.2015.20.193.6374

**Published:** 2015-03-03

**Authors:** Sancar Serbest, Haci Bayram Tosun

**Affiliations:** 1 Department of Orthopaedics and Traumatology, Faculty of Medicine, Kirikkale University, Kirikkale, Turkey; 2 Department of Orthopaedics and Traumatology, Faculty of Medicine, Adiyaman University, Adiyaman, Turkey

**Keywords:** Revision knee arthroplasty, arthroplasty complications, knee dislocation

## Image in medicine

Dislocation of revision Total Knee Arthroplasty (TKA) is a very rare, serious and difficult complication. Incidence of significant instability and dislocation following primary TKA ranged from 1% to 2% in the past ten years. This incidence has been lowered to 0.15% to 0.5% with the development of modern surgical techniques and posterior stabilized implants. It is very few reports have been published but it is not usually described in textbooks. A 72-year-old man underwent primary TKA for osteoarthritis on the left side at a private hospital 5 years ago. He had aseptic loosening and the revision was performed. He was referred with a painful locked left knee without any history of trauma (5 months post revision surgery). On clinical examination the left knee was deformity and painful and locked in 40 degree flexion (A). His radiographs taken. Radiographs showed the left knee in flexion and a posterior dislocation of the knee (B). A lower extremity CT angiography showed intact vascular structures. The patient was taken to the operation theatre for exploration and if necessary, revision. We attempted closed reduction of the dislocated knee but this was unsuccessful. Paramedian arthrotomy was performed via the previous longitudinal incision, and the knee was exposed. The mobile components were removed and replaced with a large-size tibial insert (C). The patient was doing well on his 3-month and 1-year follow up, ambulating with no assistive devices with good range of motion and no further instability and complications (D).

**Figure 1:**
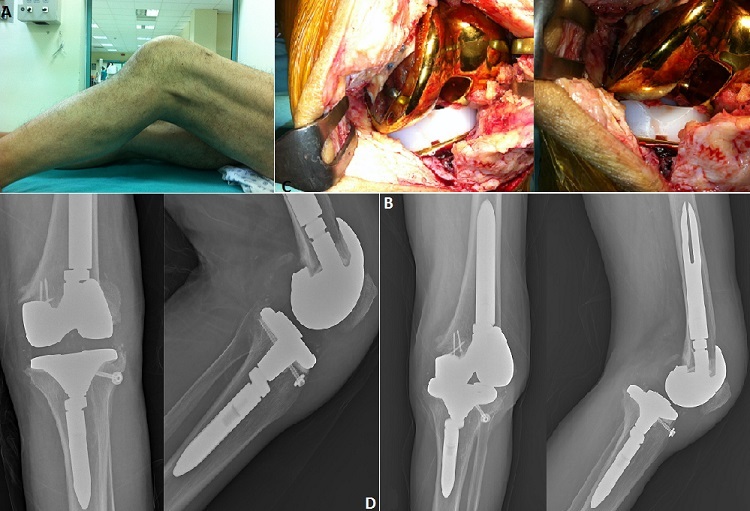
A) clinical picture after injury; B) lateral and anteroposterior radiographs of the left knee showing a posterior dislocation of the revision total knee arthroplasty; C) intraoperative findings illustrating dislocation of revision total knee arthroplasty; D) radiographs of the total knee arthroplasty after surger

